# Increased Sensitivity of CD4+ T-Effector Cells to CD4+CD25+ Treg Suppression Compensates for Reduced Treg Number in Asymptomatic HIV-1 Infection

**DOI:** 10.1371/journal.pone.0009254

**Published:** 2010-02-17

**Authors:** Georgina Thorborn, Laura Pomeroy, Heidi Isohanni, Melissa Perry, Barry Peters, Annapurna Vyakarnam

**Affiliations:** 1 Department of Infectious Diseases, King's College London, Guys' Hospital, London, England; 2 Department of Infectious Diseases, King's College London, St Thomas' Hospital, London, England; University of California San Francisco, United States of America

## Abstract

**Background:**

In HIV infection, uncontrolled immune activation and disease progression is attributed to declining CD4+CD25+FoxP3+ regulatory T-cell (Treg) numbers. However, qualitative aspects of Treg function in HIV infection, specifically the balance between Treg cell suppressive potency versus suppressibility of effector cells, remain poorly understood. This report addresses this issue.

**Methodology/Principal Findings:**

A classic suppression assay to measure CD4+CD45RO+CD25hi Treg cells to suppress the proliferation of CD4+CD45RO+CD25− effectors cells (E) following CD3/CD28 polyclonal stimulation was employed to compare the suppressive ability of healthy volunteers (N = 27) and chronic, asymptomatic, treatment naïve, HIV-infected subjects (N = 14). HIV-infected subjects displayed significantly elevated Treg-mediated suppression compared to healthy volunteers (p = 0.0047). Cross-over studies comparing Treg cell potency from HIV-infected versus control subjects to suppress the proliferation of a given population of allogeneic effector cells demonstrated increased sensitivity of CD4+CD25− effector cells from HIV-infected subjects to be suppressed, associated with reduced production of the Treg counter-regulatory cytokine, IL-17, rather than an increase in the suppressive potential of their CD4+CD25+ Treg cells. However, compared to controls, HIV+ subjects had significantly fewer absolute numbers of circulating CD4+CD25+FoxP3+ Treg cells. In vitro studies highlighted that one mechanism for this loss could be the preferential infection of Treg cells by HIV.

**Conclusions/Significance:**

Together, novel data is provided to support the contention that elevated Treg-mediated suppression may be a natural host response to HIV infection

## Introduction

A subpopulation of CD4+ T lymphocytes called Regulatory T cells (Treg cells) has attracted a significant amount of interest due to their ability to negatively regulate immune responses. In humans, this population which is CD25 positive, comprises 5–10% of normal CD4+ T lymphocytes with the majority thought to develop in the thymus soon after birth and are termed ‘natural’ Treg cells (nTeg cells) [1]–[3]. In addition to CD25, the expression of a forkhead/winged helix transcription factor called FoxP3 in thymus-derived nTreg cells is also necessary for nTreg lineage specification in both humans and mice [1]–[3]. In humans, X-linked mutations in FoxP3 alleles causes multi-organ autoimmune disease called Immunodysregulation polyendocrinopathy and enteropathy X-linked syndrome (IPEX) [1]–[4]. However, not all CD4+T cells with suppressive capacities associated with Treg function emerge from thymic development. Thus, peripheral CD4+T cells can acquire a Treg phenotype when encountering cognate or foreign antigen in the presence of regulatory cytokines such as IL-10 (Tr1) and TGF-β (Th3), and are referred to as ‘induced’ (iTreg) or ‘adaptive’ Treg cells [1]–[3]. A major limitation that remains in the Treg biology field is the isolation of functional Treg subsets with a definitive marker as traditional Treg cell associated markers are also expressed transiently on non-regulatory activated T cells (e.g., GITR, CD25, CTLA-4, FoxP3) [1]–[3]. Therefore, determining if a cell population is genuinely regulatory is contingent on a functional *in vitro* assay of T-effector cell suppression.

Treg cells have a diverse TCR repertoire, can regulate immune responses to both self and foreign antigens and initially found to be critical in maintaining self-tolerance against autoimmune disorders [1]–[3]. More recent *in vitro* studies though highlight CD25+ CD4+ Treg cells to restrain the vigour of diverse antigen-specific responses in humans, including those directed against tumours, parasitic, fungal, bacterial and viral antigens and consequently to be associated with the inability to clear infection of some pathogens [1]–[3], [5]–[6] or mount an effective immune response following immunization in *in vivo* murine model systems [7]. However, in HIV infection, Treg cells appear to play-opposing roles, contingent on disease stage. Both animal and human studies demonstrate that Treg cell numbers are elevated in the acute stage of virus infection and could dampen the virus-specific adaptive T-cell response, which may prevent effective virus clearance [8]–[11]. Thus, peripheral blood derived CD4 T cells from HIV+ subjects in the acute stage of infection depleted of autologous Treg cells proliferated more efficiently and secreted more IFN-gamma when stimulated with HIV antigens [8], [9]–[11]. However, in the chronic phase of HIV infection, although CD4+ CD25+ Treg cells have been shown to suppress both HIV-specific CD8 and CD4 T-cell functions [12]–[20], including the secretion of CD8 antiviral soluble factors [13], the presence of these cells maybe beneficial in controlling immune activation and subsequent disease progression [16], [17]–[20]. This is exemplified by an inverse correlation between Treg cell frequency and immune activation markers (e.g. CD38 and HLA-DR) or clinical markers of disease progression (i.e., low CD4 cell counts/high viral loads) [16]–[20]. Similarly, a key difference between pathogenic and non-pathogenic SIV infection in the natural host is the level of immune activation and reduced Treg cell numbers in animals infected with pathogenic SIV [11], [21]–[22]. An important question that follows and one that is poorly understood, is whether SIV, or indeed HIV infection, alter the quality of the Treg response in addition to the overall circulating numbers of these cells. This study investigates this issue. We demonstrate CD4+ CD25+ Treg-mediated suppression to be elevated, rather than compromised, in asymptomatic HIV-infected subjects with fewer absolute numbers of circulating Treg cells than control subjects. Elevated suppression was noted to be due to increased sensitivity of effector CD4 T cells from HIV-infected subjects to be suppressed, which in-turn was linked to reduced production of the pro-inflammatory, Treg counter-regulatory cytokine, IL-17 [see 23], rather than increased Treg cell potency. This study therefore provides fresh insight to how enhanced Treg-cell function may compensate for declining Treg number and thereby help maintain immune homeostasis in chronic HIV infection.

## Results

### Potency of Treg-Suppression Is Dependent on Strength of Stimulus and on Effector∶Treg Cell Ratio

**Figure 1:**
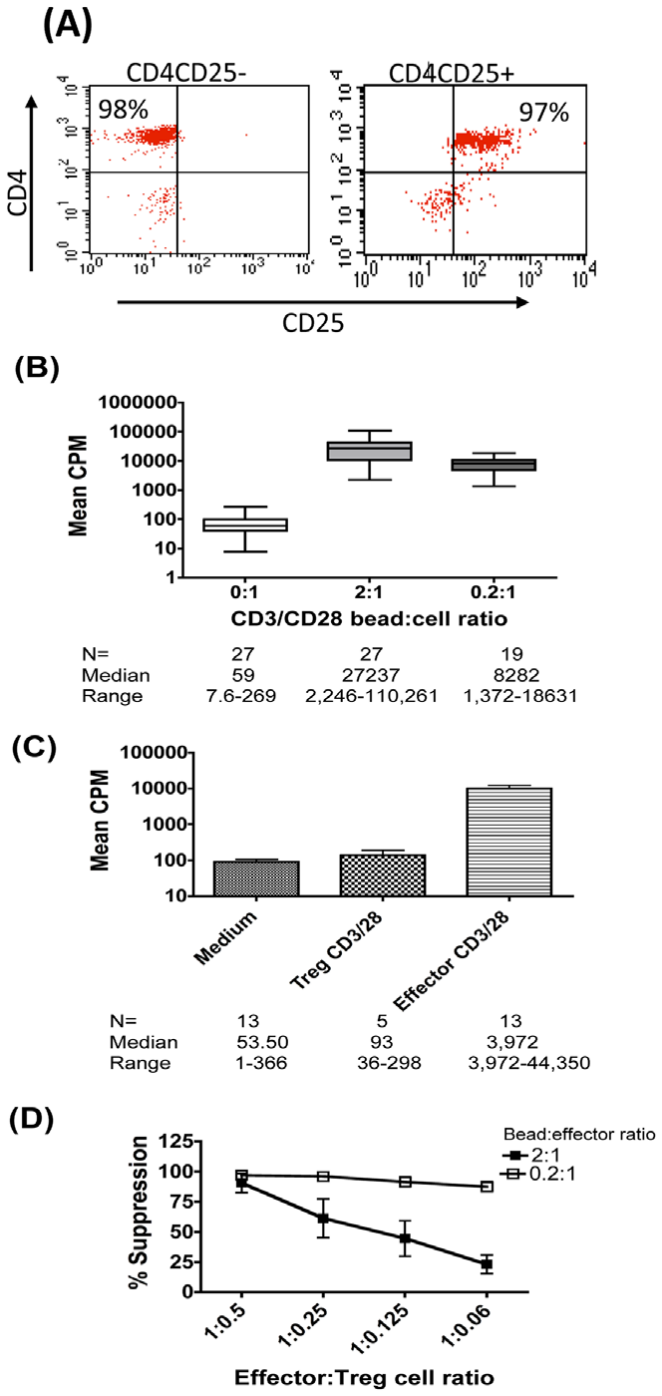
Potency of Treg-suppression is dependent on strength of stimulus and on Effector∶Treg cell (E∶T) ratio. (A) Typical FACS plot displaying purity of effector (CD4+CD25−) and Treg (CD4+CD25+) populations in healthy control subjects. (B) Mean counts per minute (CPM) (+SEM) uptake of tritiated thymidine in CD25− effector T cells from healthy controls (N = 27) stimulated with mitogenic CD3/28 beads at 2∶1 and 0.2∶1 bead: cell ratios. (C) Mean CPM (+SEM) in CD25− effectors v CD25+ Treg cells stimulated with mitogenic CD3/28 beads at 2∶1 bead: cell ratio. (D) Mean (+SEM) percentage suppression of effector cell proliferation following activation with CD3/28 beads at 2∶1 (closed boxes) and 0.2∶1 (open boxes) bead: cell ratios.

To reduce potential assay variation arising from varying ratios of memory versus naïve CD4 effector cells in the suppression assay due to the selective loss of CD4+CD45RO+ memory cells in HIV infection, we isolated >95% pure effectors and Treg cells based on differential CD25 expression from within the CD4+CD45RO+ T-cell compartment (Fig. 1A). Culture conditions were optimised to identify the minimum number of CD4+CD45RO+CD25− effector cells that consistently proliferated to mitogenic CD3/28 bead stimulation. A matrix comprising a sliding scale of effector cell number and concentration of stimulus was optimised. Figure 1B shows CD3/28 dose dependent effector cell proliferation as measured by tritiated thymidine incorporation. The mean count per minute (CPM) in cultures from 27 healthy volunteers was 27237 and 8282 at 2∶1 (high) and 0.2∶1 (low) CD3/28 bead: cell ratio respectively compared to a CPM of 59 in the no stimulation control. In contrast purified Treg cells were consistently anergic (Fig 1C). Fig 1D shows that at the higher CD3/28 stimulation dose (2∶1 bead∶effector cell ratio) percent suppression falls from an average of 90% to 23% as the effector ∶ Treg cell (E∶T) cell ratio decreases. However, when the stimulation dose was reduced, suppression levels were maintained close to 100% even at the lowest E∶T ratio tested. These data confirm the optimised suppression assay to reflect established principles of Treg function, dependent on both Treg cell number and signal strength [24]–[25].

### The Quality of Treg Cell-Mediated Suppression Is Significantly Enhanced in HIV Infection

**Figure 2:**
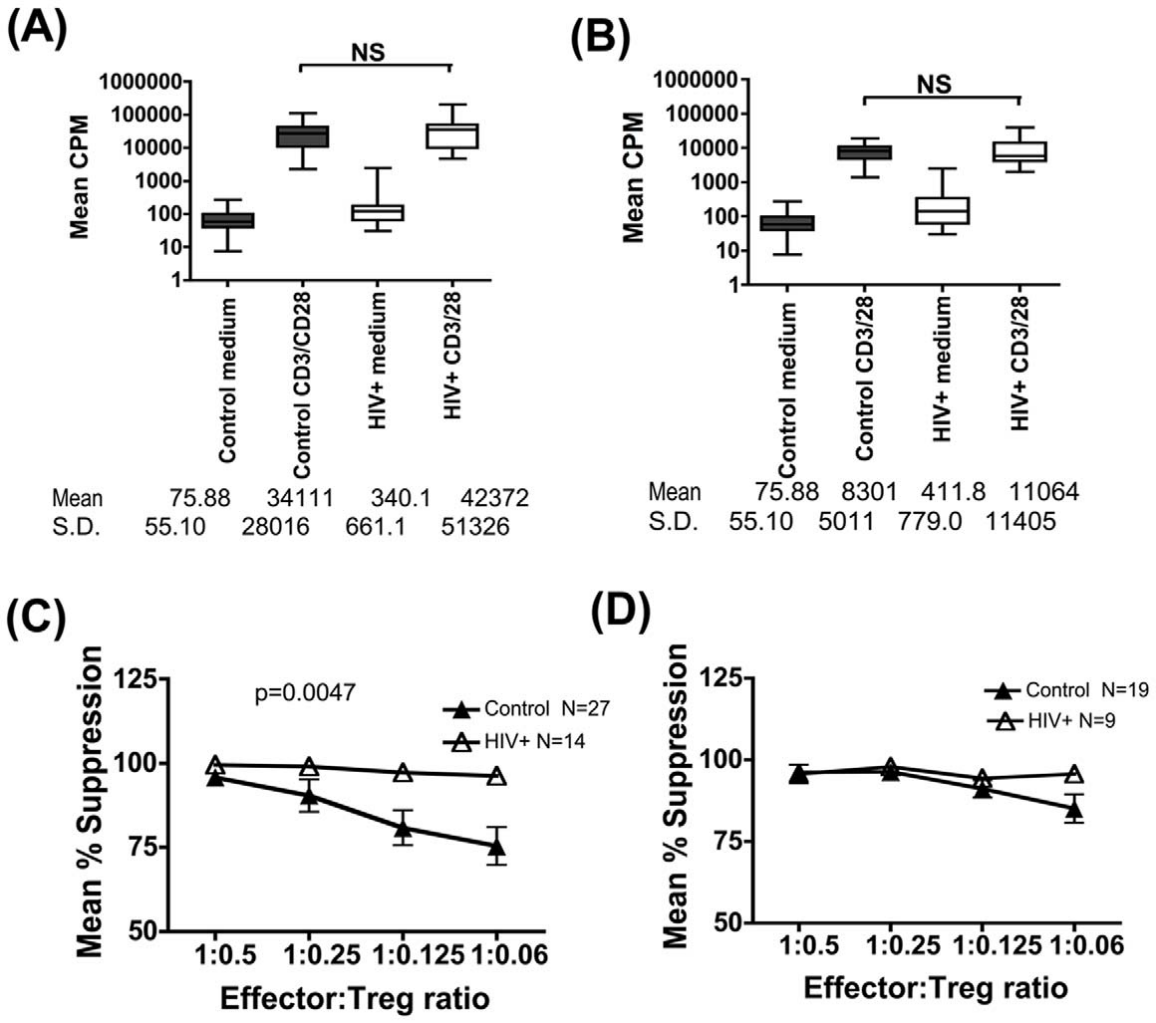
Treg cell-mediated suppression is significantly enhanced in HIV infection. 2.5×10^3^ effector T cells were cultured with two stimulation doses of CD3/CD28 coated magnetic beads, at bead∶cell ratios of 2∶1 (A) and 0.2∶1 (B). Mean CPM (+SEM) uptake of tritiated thymidine is shown. Mean (+SEM) percentage suppression of effector cell proliferation in HIV+ (open triangles) v control subjects (closed triangles) tested in parallel, following activation with CD3/28 beads at 2∶1 (N = 27 control and 14 HIV+ subjects) (C) and 0.2∶1 (N = 19 control and 9 HIV+ subjects) (D) bead: cell ratios is shown. For figures 2(C) and 2(D), the Kruskal-Wallis test was used to determine overall mean differences between groups and only the significant 2-sided p-value (Fig 2C) shown. The Mann-Whitney test was then used to compare differences between the patient and control groups at individual E∶T ratios in Fig 2C, which highlighted the following significant differences: E∶T = 1∶0.125, p = 0.026 and E∶T = 1∶0,06, p = 0.048.

The suppression assay described above was used to compare the quality of Treg function HIV infected subjects and control subjects. First, it was confirmed that effector cells from control and HIV+ subjects displayed comparable levels of proliferation, both at the high and low CD3/28 mitogenic bead∶cell stimulation ratios of 2∶1 and 0.2∶1 (Fig. 2A and 2B respectively). At the higher CD3/CD28 stimulation dose, overall suppression levels were significantly higher in cultures from HIV+ subjects versus controls (p = 0.0047,Kruskal-Wallis). Differences between the two groups were noted at the lower E∶T ratio of 1∶0.125 (97.3%±4.5 vs 80%±27.1, HIV+ v controls respectively, p = 0.026, Mann-Whitney), and 1∶0.06 (96.2%±5.4 vs 75.4%±27.6, HIV+ v controls respectively, p = 0.048, Mann-Whitney) (Fig. 2C). As would be anticipated, this gain of function effect of elevated suppression in HIV+ cultures was only evident at the high CD3/CD28 stimulation dose where suppression levels dropped in control cultures as Treg number was lowered (Fig 2C) and not at the lower stimulation dose where high levels of suppression were maintained in the control group over a wider range of E∶T ratios (Fig 2D). Thus on a per cell basis, higher Treg-mediated suppressor activity was noted in HIV+ cultures v controls. There was no apparent correlation between the potency of suppression and clinical parameters such as patients VL, CD4 count and time from diagnosis (Table 1).

**Table 1:** Summary of clinical data and Treg-mediated suppression.

Patient I.D	Sex	Age (Years)	CD4 count (Cells/ml)	Viral load (Copies/mm3)	Time since diagnosis (Years)	CD4+CD25+ FoxP3 absolute numbers	Mean suppression of effector cell proliferation at 1∶0.125	Mean suppression of effector cell single IFNγ at 1∶0.001
006	M	41	470	<50	3			
010	M	42	1065	10969	9		95.92124*	22.4*
013	F	37	362	2032	16		97.28432	
020	M	40	629	4350	12		99.21849*	34.6*
031	F	42	647	<50	5	10.10	99.33171*	15*
033	F	43	495	1573	4	2.69		
034	M	37	642	261	8	3.17		
036	M	26	501	367	3		99.75747	
037	M	53	1033	295	4	23.28		0
039	M	34	594	3572	3		99.27153*	73.9*
042	M	38	793	1692	3		99.3157	1.7
048	M	30	362	2448	7.5	8.65	99.30474*	68.7*
053	M	37	763	11224	8	10.29	99.42773*	34.9*
059	F	31	1239	124	4	49.31	93.30409*	62.5*
063	F	32	406	4109	4	2.23	98.63402	
080	M	45	431	5407	8	19.61	98.714*	40.2*
082	M	42	763	710	2			100
083	F	32	461	18779	5	11.77	99.78509	
087	M	34	540	6058	7	18.57		0
094	M	47	1199	1646	3.5		82.92561*	14.28*
096	M	45	446	<40	8			
Median		38	594	1692	5	N/A	N/A	N/A
Range		26–53	362–1238	<40–18779	2–16			
Mean		38.48	659.1	3607	6.048	17.05	97.2997	36.04
S.D.		6.516	269.9	4791	3.467	12.35	4.51	31.87
Controls								
Mean	N/A	N/A	N/A	N/A	N/A	34.03125	80.08	11.69
S.D.						18.65	27.1	16.41
P- value						0.0176	0.0286	0.041

A summary of clinical parameters of all HIV+ subjects studied along with the following functional data is summarised: (i) Absolute CD4+CD25+FoxP3+ Treg cell number and % specific Treg-mediated suppression of (ii) effector cell proliferation or (iii) single IFN-gamma expression. Effector cells in both suppression assays were stimulated with 2∶1 CD3/28 bead to cell ratio. E∶T ratio was 1∶0.125 in proliferation assay and 1∶0.001 in the IFN-gamma suppression assay. Asterik denotes HIV+ subjects who showed significantly elevated suppression compared to the mean suppression level noted in control subjects under identical culture conditions in both suppression assay. For age and gender of controls, refer to materials and methods.

### Treg Cells from HIV+ Subjects Are Potent Suppressors of Effector Cell IFN-Gamma Expression

**Figure 3:**
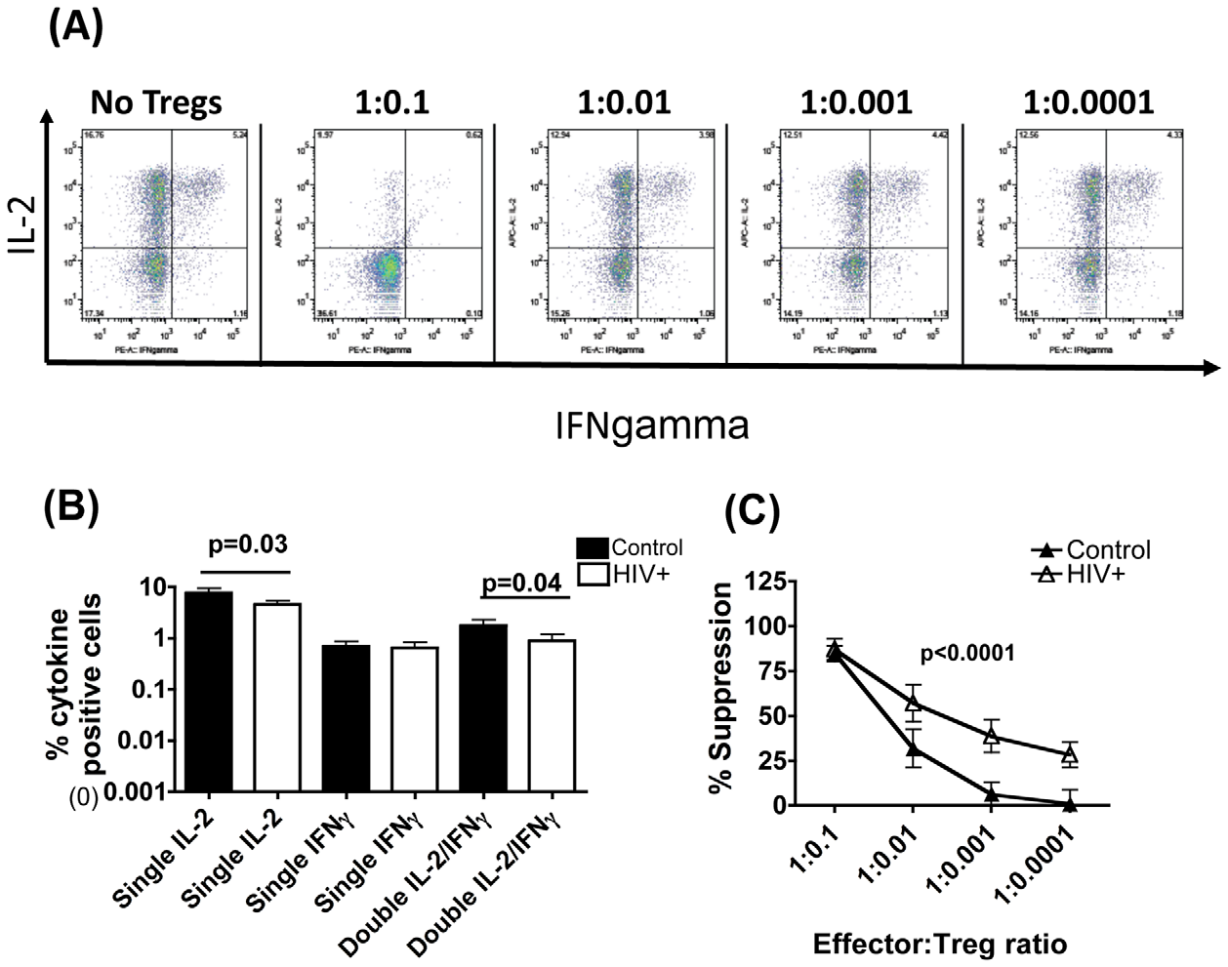
Treg cells from HIV+ subjects are potent suppressors of effector cell IFN-γ expression. 20×10^4^ effector T cells were stimulated with 2∶1 CD3/28 bead∶effector T cell ratio in the presence or absence of varying numbers of Treg cells. 5µg/ml final brefeldin A was added 2 hours after addition of stimulus and the cultures maintained for 16 hours, before washing, permeabilisation and staining for IFN-γ and IL-2. (A) Representative FACS plots show frequency of single and double IL-2 and IFN-γ+ cells in cultures with and without Treg cells. Paired t-test for differences between patients and controls in individual cytokines is shown. (B) Comparison of the frequency of single and double IL-2 and IFN-γ positive effector cells in chronic HIV+ patients (white bars, N = 18) and healthy controls (black bars, N = 13) following CD3/CD28 stimulation in the absence of Treg cells. (C) Comparison of the potency of suppression of single IFN-γ + effector cells in cultures from HIV+ (open triangle) versus control (closed triangle) subjects is shown. Unzeroed data points are plotted. For Fig 2(C), the Friedman test was used to determine overall mean differences between control (N = 13) and HIV+ (N = 13) subjects using balanced data points across all effector: target cell ratios and 2-sided p-value (p<0.0001) reported. The Mann-Whitney test was then used to compare differences between the patient and control groups at individual E∶T ratios and highlighted the following significant differences: E∶T = 1∶0.01, p = 0.04; E∶T = 1∶0,001, p = 0.0244 and E∶T = 1∶0.0001, p = 0.0188.

In addition to proliferation, Treg cells can suppress effector cell cytokine expression [1]–[3]. We examined suppression of a key effector cytokine that is widely reported to be preserved in HIV infection, IFN-gamma [26]. To study suppression of effector cells that selectively produced IFN-gamma, but not IL-2, another key cytokine that is significantly impaired in HIV infection, we employed two-colour immunofluorescence to distinguish effectors that produced one or both cytokines. In keeping with previous data from our laboratory [27] and elsewhere [26], [28] effector cells from HIV+ subjects had fewer single IL-2, and IFN-gamma/IL-2 double positive cells but preserved single IFN-gamma+ cells compared to controls (Fig. 3B). The effect of adding Treg cells was therefore measured on single IFN-gamma+ effectors following CD3/28 stimulation. Suppression levels were noted to be significantly higher in HIV+ versus control cultures, especially as Treg cell number was lowered (p<0.0001, Friedman test, Fig 3C). At an E∶T of 1∶ 0.001, mean suppression in HIV+ cultures was 46.62% versus 10.16% in controls, p = 0.0244, Mann-Whitney. At an E∶T of 1∶ 0.0001, mean suppression in HIV+ cultures was 27.93% versus 0% in controls, p = 0.0188, Mann-Whitney. Of 13 HIV+ subjects tested, 10 subjects were also tested in the proliferation assay. 9 out of these 10 subjects showed elevated suppression of both proliferation and IFN-gamma expression compared to controls, indicating good concordance between the two suppression assays (Table 1).

### Enhanced Suppression in HIV+ Cultures Reflects Increased Sensitivity of Effectors to be Suppressed Rather Than an Increase in Suppressive Potency of Treg

**Figure 4:**
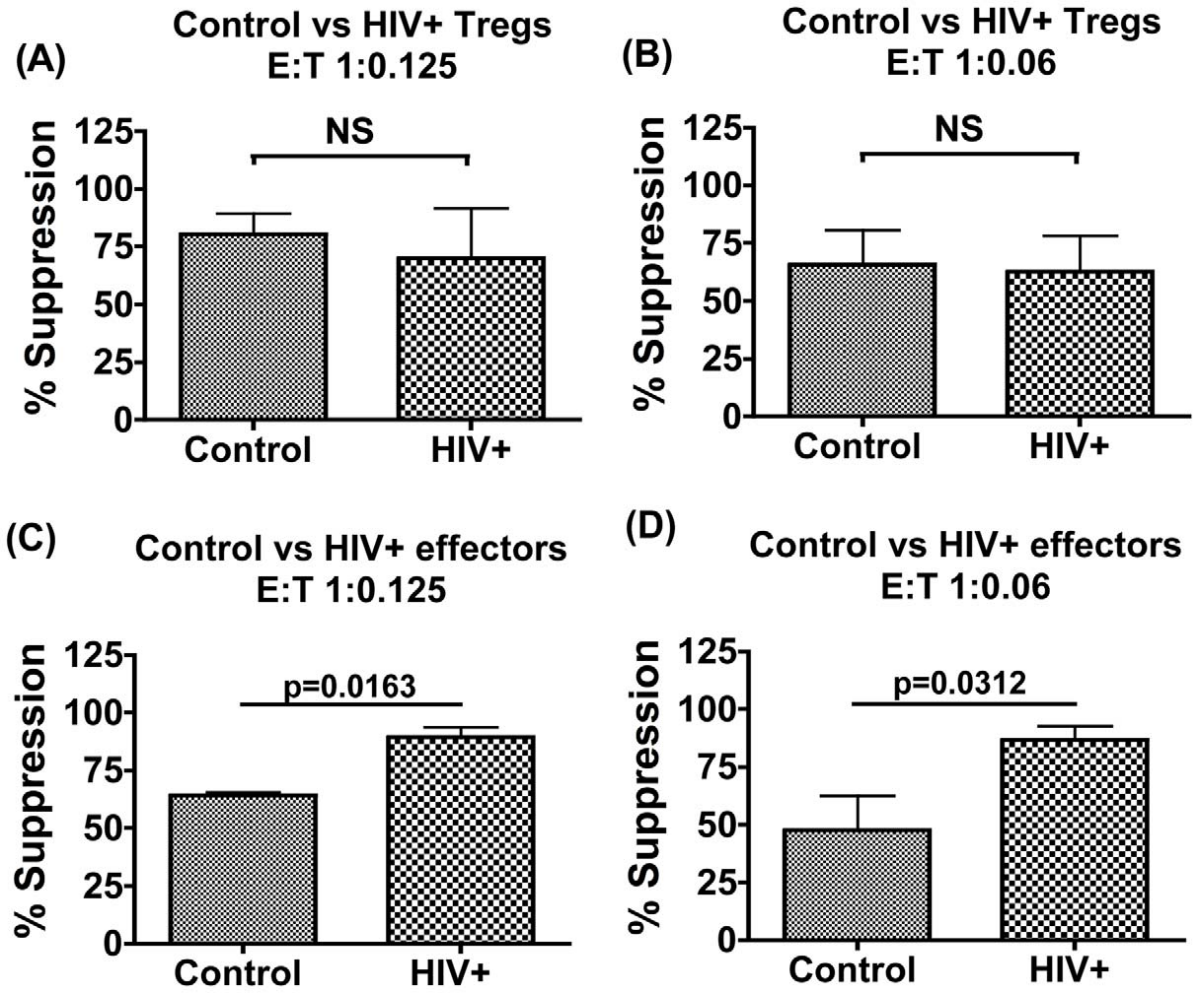
Enhanced suppression in HIV+ cultures reflects increased sensitivity of effectors to be suppressed rather than an increased suppressive potency of Treg cells. 2.5×10^3^ effector T cells were stimulated with CD3/28 beads at a 2∶1 bead: effector T cell ratio with or without the addition of Treg cells and percentage suppression calculated from three replicate wells. Comparison of the potency of Treg cells from controls versus patients to suppress a given population of allogeneic control effector cells in a paired analysis (N = 6) at two E∶T cell ratios of 1∶0.125 (A) and 1∶0.06 (B) respectively is shown. Comparison of the potency of control versus patients effector cell suppression by a given population of allogeneic control Treg cells in a paired analysis (N = 5) at two E∶T cell ratios of 1∶0.125 (C) and 1∶0.06 (D) respectively is shown.

We next dissected if enhanced suppression observed in HIV+ cultures reflected increased Treg cell potency or was due to an increased sensitivity of their effector cells to suppression. This was examined using a previously described allogeneic cross-over assay system [29]–[31]. Fig 4A, 4B compare the ability of Tregs from healthy controls versus HIV+ subjects to suppress proliferation of the same allogeneic effector cell population in a paired analysis, tested at two E∶T cell ratios of 1∶0.125 and 1∶0.06 respectively. No significant differences were observed demonstrating that Treg's from HIV+ subjects do not differ intrinsically in potency compared to control Tregs. We next compared effector cells from HIV+ subjects versus controls in their ability to be suppressed by a given population of Treg cells isolated from control subjects in a paired analysis (Fig 4C, 4D). Data shows effector cells from HIV+ subjects to be more potently suppressed than those from controls at both E∶T ratios tested. (Mean suppression at E∶T 1∶0.125 = 64.23%±2.66 in controls versus 89.53%±7.318 in HIV+ patients, and at E∶T 1∶0.06 = 47.77%±34.07 in controls versus 86.72±13.68 in HIV+ patients). Taken together, these data demonstrate increased Treg-mediated suppression in chronic HIV+ infection to be a consequence of increased sensitivity of effector cells from HIV+ subjects to be suppressed by Treg cells, rather than an increased suppressive potency of Treg cells.

### Impaired IL-17 Production by Expanded CD4+CD45RO+ CD25-Effector Cells from HIV+ Subjects

**Figure 5:**
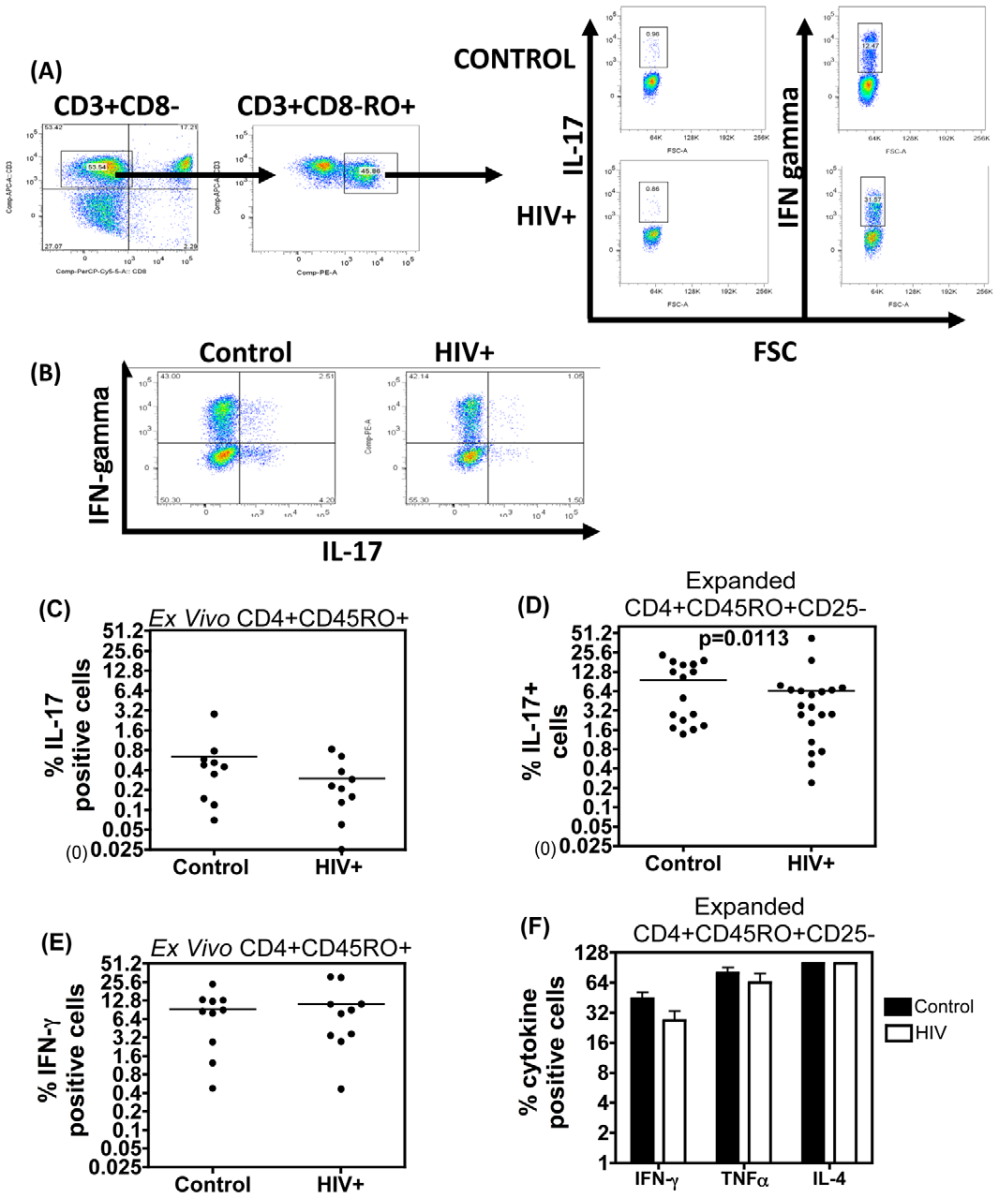
Impaired IL-17 production by IL-2 expanded effector cells from HIV+ subjects. IL-17A, IFN-γ, IL-4 and TNFα production was measured from expanded CD4+CD25− effector cell lines. Cells were expanded over six days with irradiated APC, IL-2 and purified anti-CD3 Ab and then re-stimulated for 16 hours with PMA/ION in the presence of 5µg/ml brefeldin. (A) Representative FACS plot showing gating strategy in PBMC of *ex vivo* IL-17 and IFN-γ production after 4 hours of PMA/ION stimulation from a HIV+ patient and healthy control in the presence of brefeldin A. (B) Representative FACS plots show IL-17 and IFN-γ+ CD4+CD25− cell frequency from expanded cells after 16 hr stimulation with PMA/Ionomycin in the presence of 5µg/ml brefeldin A. (C) Frequency of IL-17 and IFN-γ production from *ex vivo* CD4+CD45RO+ gated cells within PBMC (D). Frequency of total IL-17 positive cells in expanded CD4+CD45RO+CD25− effector cells from controls (N = 16) and HIV+ (N = 20) subjects. Group differences were determined by Fischer's Exact test and 2-sided p-value reported. (E) Frequency of IFN-γ, TNFα and IL-4 positive cells in expanded cell lines from 4 control (black bars) versus 6 HIV+ (white bar) subjects. (F) Differences in these individual cytokines between control v HIV+ samples was assessed by paired t-test and were non significant.

We next determined why effector cells from HIV+ patients were more susceptible to CD4 CD25+ Treg-mediated suppression. As immunosuppressive Treg-cell function shows a reciprocal relationship with the pro-inflammatory cytokine IL-17 [23], [32], [33], we determined if the increased sensitivity of HIV+ effectors to suppression was associated with reduced IL-17 production. The frequency of IL-17+ cells was measured in a standard ICS assay, with IFN-gamma serving as a positive control for activation. In keeping with other studies the frequency of IL-17+ CD4 T cells induced by TCR ligation was consistently low in *ex vivo* memory CD4 T cells within PBMCs [34]–[38], despite good induction of IFN gamma (Fig 5C). Although there was a trend for a small reduction in the percentage of IL-17+ cells in HIV+ subjects within the total memory compartment *ex vivo*, this did not reach significance for the number of samples tested within the scope of this study (Fig 5C). However expanding purified CD4+CD45RO+CD25− effector T cells with anti-CD3/IL-2 dramatically increased the frequency of IL-17+ effectors in cultures from both control and HIV+ subjects, as previously shown [34]–[35] (Fig 5D), enabling a further comparison of the capacity to induce IL-17 to be made. Figure 5D shows that control subjects segregate into two distinct groups, with 50% individuals (8/16 subjects tested) having lower frequency of IL-17+ cells than the overall group mean and 50% of subjects forming a separate group of higher IL-17+ responders. Taking this heterogeneity into account, we observed that 18/20 HIV+ individuals tested (i.e. 90%) had lower frequency of IL-17+ cells than the control group mean. Therefore the proportion of HIV+ subjects with low IL-17+ effectors was significantly higher than that of controls (p = 0.0113, Fischer's exact test), highlighting a small but significant reduction in IL-17 production by expanded effector cells in HIV+ patients. Other effector cytokines tested, IFN-gamma, IL-4 and TNF alpha (Fig 5D) did not differ significantly between the HIV+ v control groups, indicating that reduced frequency of IL-17+ effectors was unlikely to reflect global reduction of effector cytokine expression by effector cells from HIV+ subjects.

### Analysis of Treg Quantity: Reduction in Absolute Treg Cell Number in Chronic HIV Infection

**Figure 6:**
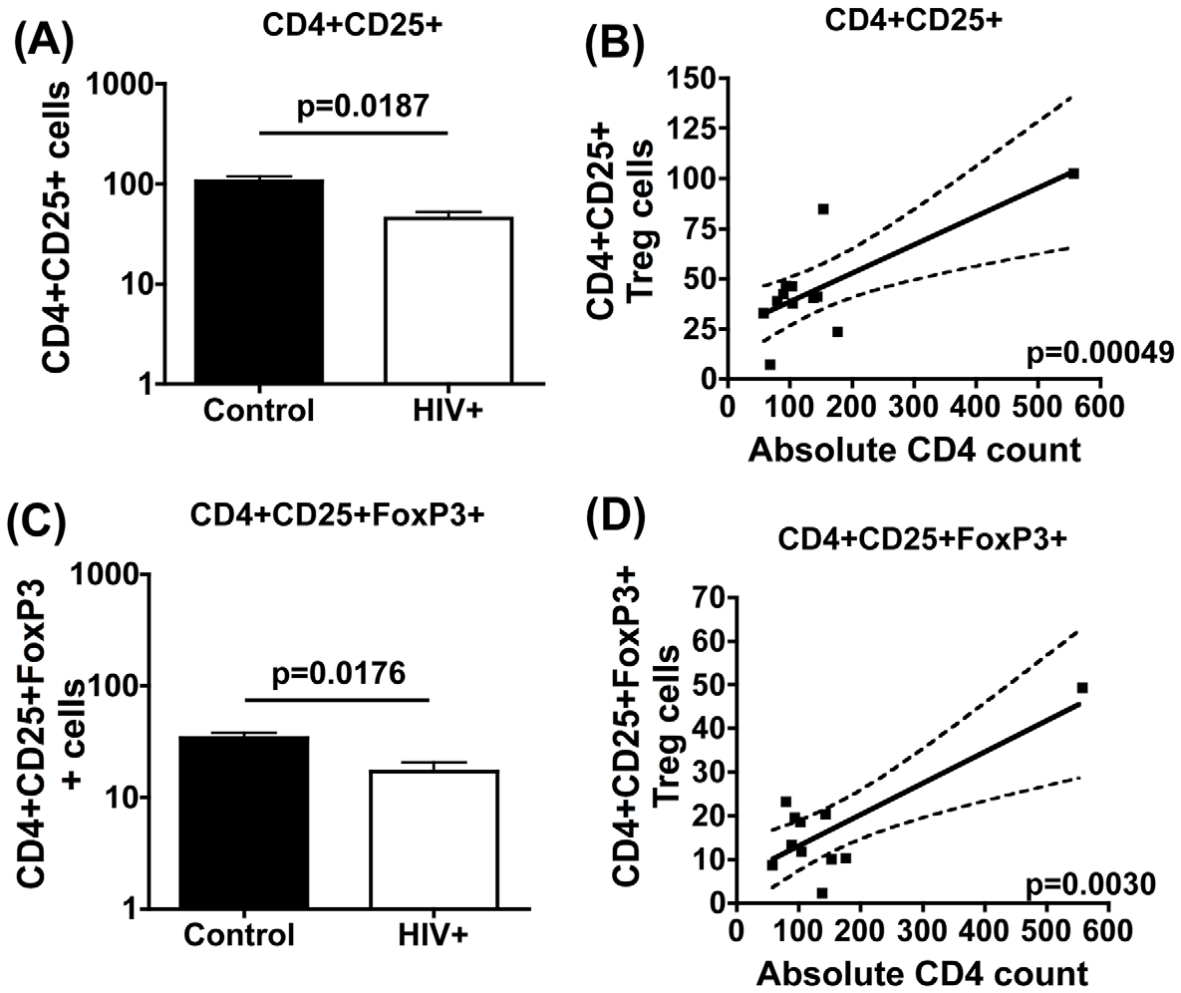
Reduction in absolute Treg cell number in chronic HIV infection. The frequency of Treg cells in HIV+ subjects defined as CD4+CD25+ (A) and CD4+CD25+ FOXP3+ (C) was first enumerated by immunofluroescence and absolute numbers of these subsets derived based on absolute CD4 T cell count of each subject. Bars represent mean values with SEM. P-values were calculated using Student's t test. Correlation between absolute Treg cell numbers and CD4 T cell count was determined using GraphPad PRISM software. (B) Correlation between CD4+CD25+ number and CD4 T cell count. (D) Correlation between CD4+CD25+FoxP3+ number and absolute CD4 cell count. P-values were calculated using Spearman's rho linear regression.

Assessing Treg cell frequency in HIV infection has to factor in the variable that CD4 T cell numbers decline following HIV infection. We therefore determined the absolute numbers of circulating Treg cells based on the CD4 T-cell count of HIV+ subjects at the time of the study. The two common markers used to define Treg cells are CD25 and FoxP3 (see Figure S1). Fig 6 shows that the absolute Treg cell number is significantly lower in HIV-infected versus control subjects. Treg cell defined as CD4+CD25+ (Fig. 6A) or as CD4+CD25+FoxP3+ (Fig. 6C) cells were both 2-fold lower in HIV+ subjects versus controls. Mean CD4+CD25+ absolute cell numbers were 45.37±25.32 cells in patients vs 96.83±71.98 in controls; p = 0.0187, and mean CD4+CD25+FoxP3 absolute numbers were 17.05± 12.35 in patients vs 34.03± 18.65 in controls; p = 0.0176. In addition, a positive correlation between CD4 count and Treg cell number with both markers was observed (Fig. 6B, p = 0.0005, and Fig 6D, p = 0.003), further confirming that Treg-cell frequency modeled CD4 T cell count in HIV infection. Of 11 subjects studied in total, 7 were also studied in the functional proliferation assay (Table 1). Data show elevated suppression in these 7 subjects with reduced Treg cell number (Table 1).

### *In Vivo* Treg Number Decline May Reflect Increased Susceptibility of Treg Cells to HIV-1 Infection

**Figure 7:**
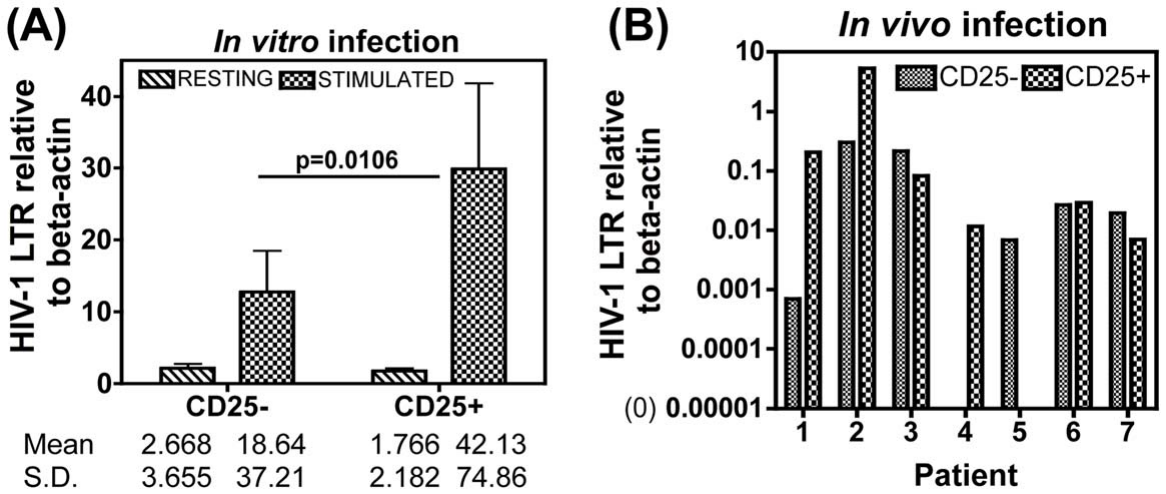
Increased susceptibility of Treg cells to *in vitro* HIV-1 infection. Purified CD4+CD45RO+CD25+ Treg cells and CD4+CD45RO+CD25− effector T cell populations from 12 healthy volunteers were infected with the HIV-1 X4 NL4-3 strain (MOI 0.1) and cultured either in IL-2 alone or stimulated with CD3/CD28 beads at 2∶ 1 bead: effector T cell ratio. 4 days later cells were harvested and HIV-1 LTR DNA levels determined by qPCR relative to the housekeeping gene beta-actin. (A) Relative copy number (RCN) of HIV-1 LTR copies in resting versus stimulated Treg and effector cells is shown in a paired analysis. (B) Paired analysis of RCN of HIV-1 LTR from stimulated Treg and effector cells isolated from 7 HIV+ subjects. For Fig 7(A), group comparisons by Kruskal-Wallis non-parametric test resulted in a 2-sided p-value <0.0001. Paired t-test was then used to compare CD25− v CD25+ samples; significant difference was noted between stimulated CD25− v stimulated CD25+ samples, p = 0.0106.

We next examined whether the loss in Treg cell numbers may be due to this subset being preferentially targeted by HIV compared to the CD25− effector population. An *in vitro* infection assay was used to determine the intrinsic capacity of these two subsets to HIV infection. Isolated Treg and effector T cell populations from healthy volunteers were infected with an X4 HIV-1 strain and productive infection measured 4 days later in cells that were left un-stimulated or stimulated with CD3/CD28. Consistent with established data that T-cell activation promotes HIV infection, we observed significantly higher HIV-1 DNA in activated versus un-stimulated cells, whether they were effector or Treg cell populations (7 and 24- fold higher infection in effector and Treg cells compared to unstimulated counterpart cells respectively (Fig. 7A). Furthermore, activated Treg cells had 2-fold significantly higher levels of HIV DNA than counterpart effector cells from the same donor (Fig. 7A) (overall group differences by Kruskal-Wallis was p<0.0001 and paired t-test of stimulated CD25− v CD25+ cells was p = 0.0106). Unstimulated Treg versus effector cells did not differ in their infection levels (Fig. 7A). These data highlight that T-cell activation promotes preferential HIV infection of Treg cells compared to effector cells, despite the Treg population being anergic to CD3/28 stimulation (see Fig 1 for proliferation). To investigate whether this was reflected *in vivo*, we isolated circulating CD4+ CD25+ Treg and CD25− effector cells from HIV+ subjects and determined endogenous HIV infection levels in these subsets before and after CD3/28 stimulation. A significant level of inter-donor variation was observed, with 3/7 individuals tested having higher HIV-DNA in Treg compared to effector cells (Fig 7B, Pt 1,2,4), whilst a further 3 subjects showed the converse (Pt 3,5&7), and one patient (Pt 6) showed no difference in infection levels between the two subsets. Taken together therefore, there was no clear evidence of preferential infection of CD25+ Treg cells *in vivo*, despite *in vitro* infection studies highlighting this subset to be intrinsically more susceptible to HIV infection.

## Discussion

The present study was designed to probe the quality of CD25+ Treg cells in chronic HIV infection. Understanding precisely how Treg cell function may be altered in HIV-infected subjects is of importance in the context of determining if this increasingly important subset of CD4 T cells represents a reasonable target for immune-based therapy in HIV infection, and if such therapy would be appropriate at all stages of HIV disease. Animal studies demonstrate that cell-based therapy involving direct injection of Treg cells ameliorates a wide range of experimental models of inflammatory and autoimmune diseases [1]–[3]. The above question is particularly pertinent in HIV infection where Treg cells may play opposing roles, being associated with detrimental outcome in the acute stage by suppressing HIV-specific adaptive immune responses [8], [9]–[11] but beneficial in the chronic stage by controlling excessive immune activation [16]–[20]. These effects of Treg cells have been linked to fluctuating Treg cell numbers rather than altered Treg potency; indeed a number of studies have shown Treg suppressive potential to be preserved in both acute [8] and chronic HIV-1 infection [12]–[20]. One study by Kinter et al [12] described lymph node CD25+ Treg cells to display higher suppressive activity than peripheral blood Treg cells from the same donor, irrespective of patients VL or CD4 count, indicating that Treg cells with the highest suppressive potential may reside in sites of ongoing virus replication. However, a comparative analysis of Treg potency in HIV+ versus control subjects and underlying mechanisms has not been reported. Our study addresses this issue and both confirms and extends previous data [8], [12]–[20], by demonstrating Treg-mediated suppression of autologous effector cell proliferation and IFN gamma expression, to be not just preserved, but significantly elevated in asymptomatic chronic HIV-infected- versus control–subjects. Interestingly, suppression of IFN gamma was noted in HIV+ subjects even at the lowest E∶T ratio 1∶0.0001 (1 Treg per 10000 effectors) (Fig. 3C). A possible explanation for this heightened suppression may be linked to our data discussed below, showing that effector cells from HIV-infected subjects are more susceptible to Treg-mediated suppression. Secondly, it is possible that the mechanism of suppression in HIV+ cultures may not be exclusively through a classical contact-dependent mechanism. Both these mechanisms potentially require fewer Treg cells than classical contact-dependent suppression and are the subject of on-going investigation.

We present novel data on the mechanisms underlying the phenomenon of enhanced Treg-mediated suppression in HIV infection, using methodology previously used to probe Treg function in autoimmune disorders [29]–[31]. In mixed cultures of Treg and effector CD4 T cells, net Treg-mediated suppression reflects intrinsic suppressive potential of Tregs as well as effector cell suppressability. The allogeneic cross-over assay has been employed successfully to dissect these two possibilities by separately testing effector and Treg cells from a given donor to be suppressed or to suppress respectively a given population of Treg v effector cells from a healthy allogeneic donor [29]–[31]. The advantage of this assay is that it enables a functional comparison to be made of Treg cells and effectors in control versus disease subjects. In lupus nephritis patients, this assay highlighted the absence of functional changes to Treg or effector cells in disease [31]. In type 1 diabetes, this assay demonstrated that reduced Treg-mediated suppression in disease was due to the effectors being more resistant to suppression, rather than the presence of defective Treg cells [29]. This study demonstrates that the phenomenon of elevated Treg-mediated suppression in HIV infection is not due to increased potency of Treg cells, rather it reflects increased sensitivity of CD4+ CD25− cells from HIV-infected subjects to be suppressed by Treg cells. These observations were consistent in samples taken from multiple patients' and controls tested in parallel against multiple allogeneic donor cell populations (Fig 4A–D).

Given the reciprocal relationship between Treg cells and the key pro-inflammatory cytokine, IL-17 [23], we explored if reduced IL-17 production by effector cells from HIV+ subjects could be one mechanism for their increased sensitivity to Treg-mediated suppression. Other recent data highlight loss of IL-17+ CD4 T cells, both in HIV [39]–[40], and in pathogenic SIV infections [39], [41]–[42]. IL-17+ CD4 T cell frequency tends to be higher in the gastrointestinal tract than blood and these cells appear to be reduced in HIV infection [39]. Loss of blood IL-17+ cells has also been noted in one study in HIV+ subjects as measured by an ELISPOT assay [40]. However, using the ICS assay in this study, only a small reduction in IL-17+ numbers in HIV+ subjects v controls was observed, which did not reach statistical significance within the scope of this study. On the other hand, evidence is provided that the capacity of CD4+CD45RO+CD25− expanded effector cells to produce IL-17 upon re-stimulation in 18/20 HIV+ subjects tested was significantly reduced than that of control subjects (Fig 5B). Studies that have examined if Th17+ cells are preferentially infected and thereby lost in HIV infection do not however support this contention [39], [43]. Another mechanism for reduced Th17 cell frequency in HIV infection may be due to this subset being highly prone to cell death. This would be evolutionarily consistent with a fundamental role of Th17 cells in immunity, whereby expansion of this subset, like Th1 cells, is under tight control through AICD and/or apoptosis [44], or IDO-mediated tryptophan-deprivation [45] or galectin-1 signalling [46]. It appears that in pathogenic SIV infection, Treg/Th17 balance is perturbed in the acute stage of infection, with loss of IL-17 production associated with increased Treg frequency correlating with detrimental outcome [41]. Taken together, it is clear that further studies are needed to elucidate the significance of IL-17 in HIV infection. It is important to establish whether or not a significant reduction in the capacity of effector cells to produce IL-17 upon activation through the T-cell receptor complex can be validated in cells isolated *ex vivo* from blood using larger sample numbers than that used in this study and if a reduction of these effectors translates to overall reduction in IL-17 affecting Treg/Th17 balance or is compensated for by IL-17 production by other cell types. In addition, a direct role for reduced IL-17 contributing to increased susceptibility of effectors to suppression will require additional investigation using endogenous IL-17 knock-down and/or reconstitution strategies. Ongoing studies aim to address these questions to better understand why effector cells from HIV+ subjects are more prone to Treg-mediated suppression.

Our data on Treg cell numbers is consistent with a number of other studies showing a fall in circulating Treg number in chronic HIV infection [16], [18], [19], [47]. Two mechanisms may account for this phenomenon: (i) preferentially HIV infection [48]–[51], and/or (ii) redistribution of Treg cells from blood to sites of active virus replication [12], [15], [47], [52]. A number of *in vitro* studies show preferential infection of Tregs with R5/X4 strains [48]–[51], which has been linked to this subset expressing high CCR5 [49] and/or FoxP3 serving to directly active the HIV-1 LTR [49], [50]. In addition, gp120/CD4 interactions may promote the activation, expansion and suppressive potential of Treg v effector cells [15], [53], [54], by up-regulating expression of the anti-apoptotic factor BCL-2, thereby prolonging cell survival and infection [15], [54]. We also demonstrate that Treg cells are intrinsically more capable of supporting HIV infection *in vitro* compared to CD4+CD25− counterparts. However, we did not find clear evidence of preferential HIV infection of circulating Treg cells in HIV+ subjects. One reason for this discrepancy may be linked to HIV-infected Treg cells not surviving following HIV infection *in vivo* or trafficking to tissue sites, as previously shown [12], [15], [47], [52], thereby confounding *ex vivo* analysis of virus compartmentalisation in circulating Treg cells isolated from HIV+ subjects. In support of this contention, Ji & Cloyd (2009) [54] observed that *in vitro* infection of Treg cells leads to the up-regulation of the homing receptor CD62L and integrin alpha4beta7, and upon injection of HIV and mock infected Treg cells into SCID mice, infected Tregs could be found in higher numbers in lymph nodes and lymphoid tissue. This is also corroborated by data showing correlation of virus load in HIV-infected subjects with Treg cell increase in the gut [52], tonsillar tissue [15], and lymph nodes [47], and normalisation after initiation of HAART [47], [52]. Further studies to elucidate the cellular and molecular mechanisms of Treg versus effector cell function in HIV infection combined with animal models that allow the manipulation of Treg cell number *in vivo*, may give us a better understanding of the real contribution Treg cells play in HIV disease progression.

## Materials and Methods

### Subjects

Peripheral blood samples in EDTA from HIV-infected subjects were obtained from Guy's and St. Thomas' Charity Infectious Disease Biobank facility. This study was approved by an independent ethics committee [Ethics reference 06/Q1909/48] and informed consent obtained from all patients. A total of 19 HIV-infected subjects were studied (see Table 1 for clinical details). All patients enrolled in this study were diagnosed as being HIV infected for at least two years prior to the study, they were treatment naïve with a stable CD4 count, as measured on at least two occasions (from time of diagnosis and at six-twelve monthly intervals) prior to sampling. Median infection time in years since diagnosis was 5 (range 3–16 years). Median CD4 count was 594 cells/mm^3^ (range 362–1239) and therefore within the normal range of CD4 count (300–1500 cells/mm^3^) observed in healthy controls [55]. 3/19 patients studied had undetectable viral loads (VL). Median VL was 1692 RNA copies/ml (range<40–18,779). Control HIV seronegative blood samples were either purchased from the National Blood Transplantation Service at St George's Hospital Tooting, UK or obtained through the Infectious Disease Biobank and tested in parallel with samples from HIV+ subjects. Where information was available, control subjects were matched as closely as possible in terms of age with that of patients, and attempts were also made to match these groups in terms of gender. The mean and median ages of the HIV positive volunteers were 38.5 years and 38 years respectively and for the healthy controls were 28.6 years and 26 years respectively. The gender matching proved practically difficult because of the nature of the gender of the volunteers who came forward; whereas 71.5% of the HIV positive participants were male, the corresponding figure for the healthy volunteers was 12.5%.

### Cell Separation

Peripheral blood mononuclear cells (PBMC) were isolated by density gradient centrifugation (Lymphoprep: Axis-Shield PoC AS, Oslo, Norway). CD4+CD25− and CD4+CD25+ cell populations were isolated using Dynabeads T regulatory cell isolation kit (Invitrogen, UK) following manufacturers instructions. Briefly, CD4+CD45RO+ T cells were isolated from PBMC by negative selection. 10^8^ PBMCs were first incubated for 20 minutes at 4°C with 200ul of an antibody cocktail directed against non-CD4 T-cell PBMC subsets plus purified CD45RA antibody 10^8^ PBMCs/1µl (eBioscience, San Dieago, CA, USA). Excess antibody was washed off and cells resuspended at 10^8^/2ml PBMCs in PBS/2%HS and 10^8^/1ml PBMC of immunomagnetic beads were added at room temperature for 20 minutes. Cells bound to the beads were removed on a magnet and the non-bead fraction confirmed by immunostaining to be >95% CD4+CD45RO+. CD25+ cell fraction (Treg) was subsequently isolated from CD4+CD45RO+ cells using 10^6^/10µl cells of mouse anti-human CD25+ positive selection beads at 4°C for 25 minutes. Tregs were detached from the positive selection beads at room temperature for 45 minutes using Detach-a-bead©. CD25− fraction (effector population) was isolated by negative selection from the remaining CD4+CD45RO+ fraction with the addition of 10^6^/50µl cells of CD25+ positive selection Dynabeads at room temperature for 40 minutes. The purity of each population was examined using monoclonal antibodies (mAbs) against CD4, CD45RO, CD25, and FoxP3. Purities were routinely >95%.

### Treg Suppression Assay as Measured by Suppression of Effector Cell Proliferation

All assays were carried out in RPMI-1640 Glutamax 25mM HEPES media (Invitrogen, Paisley, UK), 10% human AB serum (Lonza, Sweden), and supplemented with 20µg/ml Gentamycin (Sigma-Aldrich, UK). Suppression assays were conducted by plating 2.5×10^3^ CD4+CD25− effector cells per well in a 96 well plate with varying ratios of CD4+CD25+ Treg cells (Effector: Treg ratio), 1∶0.5, 1∶0.25, 1∶0.125, 1∶0.06, serially diluted to 1∶0.003). Cells were stimulated with two ratios of Dynal anti-human CD3/CD28 coated magnetic beads (bead: effector cell ratio, 2∶1 and 0.2∶1) (Invitrogen, Paisley, UK). Assays were set up in triplicate. Cultures were maintained for 5 days. Each well received 0.5µCi of (^3^H)-thymidine (GE Healthcare, UK) for the last 16 hours of culture. Cell proliferation was assessed by uptake of (^3^H)-thymidine by processing samples through a cell harvester (Perkin-Elmer, UK). 3H- thymidine uptake was measured as counts per minute (CPM) on a Betacounter (Perkin-Elmer, UK). Percentage suppression was calculated as 100-(counts per minute (cpm) of co-cultures/cpm of effectors alone) ×100).

### Treg Suppression Assay as Measured by Suppression of Effector Cytokine Expression

20×10^3^ CD4+ CD25− effector cells were cultured in 96-well plates with varying ratios of CD4+CD25+ Tregs (effector: Treg ratios, 1∶0.1, 0∶0.01, 1∶0.001 and 1∶0.001) and stimulated with 2∶1 (bead: effector cell) anti-human CD3/CD28 coated magnetic beads (Invitrogen, Paisley, UK). 5µg/ml of Brefeldin A (Sigma-Aldrich, UK) was added 2 hours post-stimulation. Cultures were maintained for 16 hours before intracellular staining (ICS) for Interferon-gamma (IFN-gamma) and Interleukin-2 (IL-2). ICS was performed once the cells had been washed twice with cold wash buffer (PBS 2% FCS, Invitrogen, Paisley, UK). A cell fixing and permeabilisation kit (AbD Serotec, Oxford, UK) was used according to manufacturer's instructions. Briefly, cells were fixed using 100µl of solution A (fixation buffer) for 20 minutes at RT, then washed and resuspended in 45µl of solution B (permeabilisation buffer) plus 5µl FCS (Gibco, Invitrogen, UK) with 5µl each PE-labelled anti human IFN-gamma, APC-labelled anti human IL-2, or the appropriate isotype control mAbs for 60 minutes at room temperature. Samples were washed ×3 and resuspended in fixation buffer (PBS, 4% formaldehyde) and acquired using BD FACSDiva software (Becton Dickson, UK) on a Becton Dickson FACSCanto II fluorescence activated cell sorter (Becton Dickson, UK). A minimum of 30–50,000 events were acquired for analysis. Analysis was performed using FlowJo software (Treestar Inc., Ashland, OR, USA). Percent suppression was calculated as detailed in section above.

### IL-17 Detection

For *ex vivo* detection of IL-17 in PBMCs, PBMCs were stimulated with 10ng/ml PMA and 1µg/ml Ionomycin (Io) (Sigma-Aldrich, UK) for 4 hours in the presence of 5µg/ml Brefeldein A. ICS staining was performed as described above following surface staining with CD3 (APC labelled, BD Pharmingen), CD8 (PE-CY5 labelled Pharmingen) and CD45RO antibodies (PE labelled, BD Pharmingen). For detection of IL-17 in expanded effector cells 0.5×10^6^ purified CD4+ CD45RO+ CD25− effector cells were cultured at a ratio of 0.5∶1 with irradiated mixed donor PBMCs in the presence of 30IU/ml IL-2 and 1µg/ml purified soluble anti-human CD3 (eBioscience, UK). On day 6 post expansion, effector cells were re-stimulated overnight with 10ng/ml PMA (Sigma-Aldrich,UK) and 1µg/ml Ionomycin (Sigma- Aldrich,UK) in the presence of 5µg/ml Brefeldin A (Sigma- Aldrich,UK). IL-17 (FITC labelled IL-17A, eBioscience) and IFN-gamma (PE-labelled), IL-4 (PE-labelled), TNFα (FITC-labelled) cytokine measurement was performed as described above by a standard ICS assay.

### Enumerating Treg Cells by Cell Surface Markers

The frequency of Treg cells was determined using a combination of established markers in purified PBMC fractions. FoxP3 expression was determined using APC anti-human FoxP3 staining set (Clone PCH101, eBioscience, San Dieago, CA, USA) as per manufacturer's instructions. Briefly, after washing cells with cold wash buffer, (PBS Gibco Invitrogen, UK, 2% FCS Gibco Invitrogen, UK) cells were first surface stained with FITC-labelled CD4 (clone SK3 BD Pharmingen, UK) and PE-labelled CD25 (clone MEM-181, AbD Serotec, Oxford, UK) at 4°C for 30 minutes. Cells were then washed and fix/permeabilised at 4°C for 60 minutes using Fix/Permeabilization concentrate and diluent©. APC-labelled FoxP3 (clone PCH101, eBioscience, San Dieago, CA, USA) was incubated for a further 30 minutes. Flurochrome conjugated isotype control antibodies were included to define specific staining. Samples were acquired on Becton Dickson FACs Calibur machine and data analysed using CellQuest software. Lymphocytes were gated based on FSC/SSC and Treg cell markers CD25 and FoxP3 were determined within the CD4 gate. Absolute numbers of Treg cells were determined as the percentage of cells staining for defined Treg cell markers multiplied by the absolute CD4 T-cell count of the patient at the time of sampling.

### HIV-1 Virus Stocks

Full length HIV infectious virus was produced by standard transient transfection of 293T cells with purified proviral DNA encoding the HIV-1 molecular clone NL4-3 (kind gift of M. Malim). 293T cells were grown in D-MEM (Gibco, Invitrogen, UK) 10% FCS (Gibco, Invitrogen, UK) 20µg/ml Gentamycin (Sigma-aldrich, UK). Transfection was performed using Fugene6 transfection reagent (Roche, UK). Culture supernatant was harvested 72 hours after transfection, clarified by centrifugation, and stored at −80°C. Negative control included virus-free culture supernatant from 293T cultures. Virus stocks were standardised on the basis of HIV-1 Gag p24 concentration measured by ELISA (NCI Frederick, HIV-1 p24 Antigen Capture Assay Kit) and by assessing virus titre in a standard infectivity assay using CEM G37 indicator cells which express green fluorescent protein (GFP) upon productive infection (kind gift Dr P Kellam, University College London). The number of infectious virus particles per ml was determined based on GFP expression induced per ml of virus stock [56].

### *In Vitro* HIV Infection

CD4+CD25− effector and counterpart CD4+CD25+ Treg cell populations were isolated from healthy HIV uninfected control volunteers. Equivalent numbers of effector and Treg cells were cultured with serial dilutions of NL4-3 virus stock, starting at a multiplicity of infection (MOI) of 0.1 based on virus stock titre. Effector and Treg fractions were cultured separately either with or without stimulation with anti human CD3/CD28 coated-magnetic for 4 days. Magnetic anti-CD3/CD28 coated beads (Dynabeads, Invitrogen, UK) were removed by placing on a magnet before cells were washed twice in cold PBS (Invitrogen, UK), pelleted and stored at −80°C until DNA extraction.

### Quantitative Real-Time PCR for HIV DNA

DNA was extracted using Qiagen DNeasy kit as per manufacturer's instructions. Briefly, cell pellets were resuspended in 200ul PBS (Invitrogen, UK), 20µl of Proteinase K solution, and then lysed in 200ul of lysis buffer at 56°C for 10 minutes. 200ul of 100% ethanol was then added and spun in a DNeasy spin column to retain DNA. After two wash steps, DNA was eluted in 80ul of kit elution buffer and stored at −20°C until analysis. For ex vivo HIV LTR detection from HIV-infected patient blood, extracted DNA had to be amplified first using a complete whole genome amplification kit (Sigma-Aldrich, UK) due to small cells numbers. HIV LTR DNA was measured relative to the house-keeping gene beta-actin using Quantitect SYBR green PCR kit (Qiagen, UK). All assays were performed on an ABI Prism 7000 machine (Applied Biosystems, CA, USA). Primer sequences for HIV-1 LTR detection [57] by SYBR green was as follows: L2: CTGTGGATCTACCACACACAAGGCTAC. L3: GCTGCTTATATGTAGCATCTGAGGGC.

Cycling conditions were 40 cycles as follows: 50°C for 2 mins, 95°C for 15 mins, 95°C for 25 seconds, 60°C for 30 seconds, then 72°C for 30 seconds. A disassociation step was introduced. Data was aquired on a SDS2.3 (Applied biosystmes, CA,USA) and analysed relative to beta-actin. Relative copy number was calculated by first determining the delta ct (gene of interest minus housekeeping gene) and then using the equation, relative copy number (RCN) = POWER(2,-delta).

### Statistical Analysis

Statistical analysis was performed using Graphpad PRISM software (Graphpad Prism Inc., version 4, CA, USA). When more than two groups were compared, an ANOVA was performed to assess overall variation. Where the ANOVA indicated a significant (p<0.05) difference, further tests were performed to determine where the differences lay. For comparison of two groups, either a non-parametric Mann-Whitney test or a paired t-test for paired samples was employed. All p-value reported are two-sided.

## Supporting Information

Figure S1 (0.16 MB TIF)
