# Supplementary figures and images for: Increased Sensitivity of CD4+ T-Effector Cells to CD4+CD25+ Treg Suppression Compensates for Reduced Treg Number in Asymptomatic HIV-1 Infection
Source: PLoS One. 2010 Feb 17;5(2):e9254. doi: 10.1371/journal.pone.0009254 (PMC2822868; doi:10.1371/journal.pone.0009254)

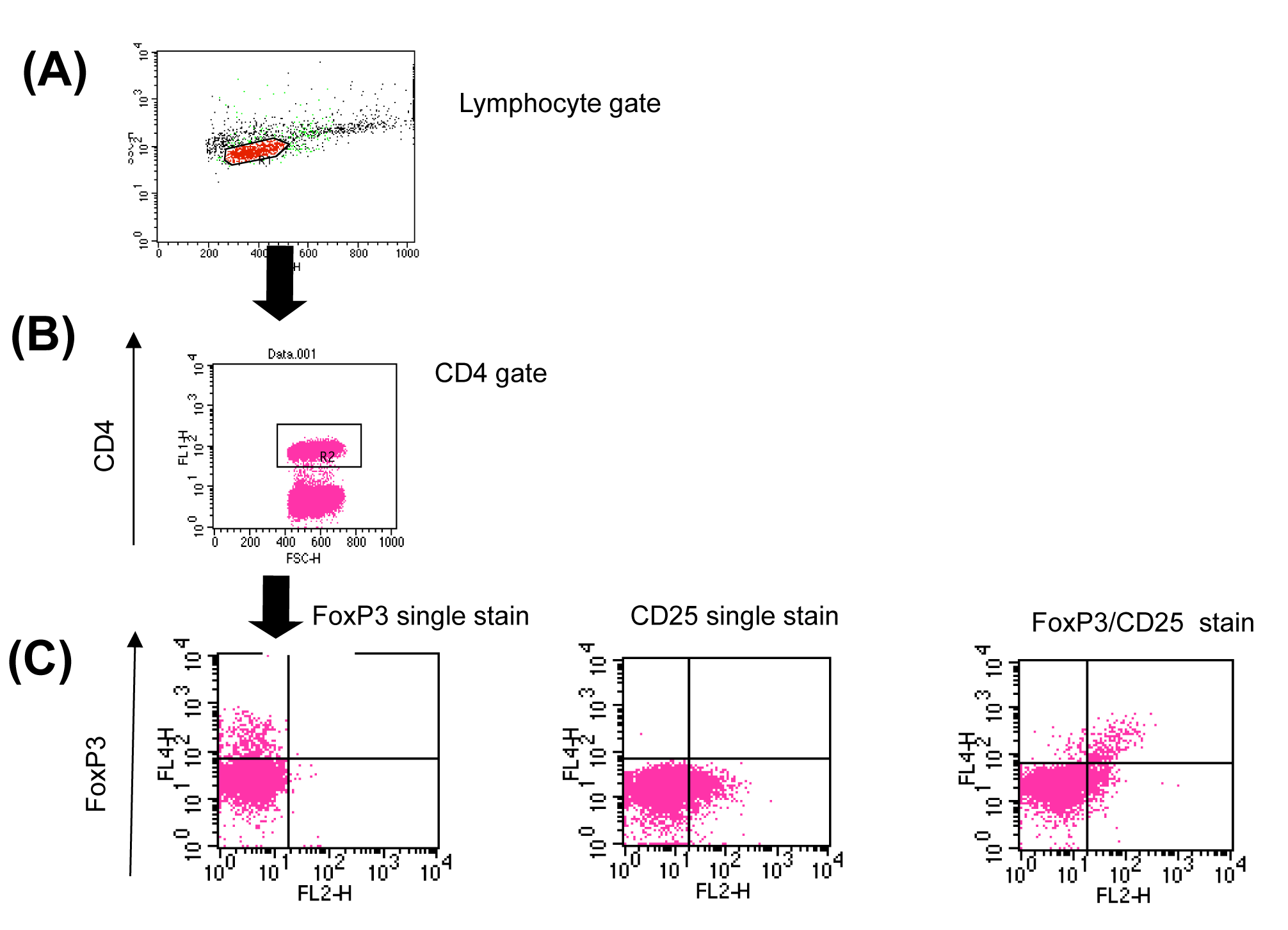

Supplement: Figure S1 — (0.16 MB TIF) [file pone.0009254.s001.tif]
